# Introgressive Hybridization and Hypoxia Adaptation in High-Altitude Vertebrates

**DOI:** 10.3389/fgene.2021.696484

**Published:** 2021-06-22

**Authors:** Jay F. Storz, Anthony V. Signore

**Affiliations:** School of Biological Sciences, University of Nebraska, Lincoln, NE, United States

**Keywords:** adaptation, altitude, elevation, HIF, hypoxia, *EPAS1*, hemoglobin, introgression

## Abstract

In natural populations of animals, a growing body of evidence suggests that introgressive hybridization may often serve as an important source of adaptive genetic variation. Population genomic studies of high-altitude vertebrates have provided strong evidence of positive selection on introgressed allelic variants, typically involving a long-term highland species as the donor and a more recently arrived colonizing species as the recipient. In high-altitude humans and canids from the Tibetan Plateau, case studies of adaptive introgression involving the HIF transcription factor, *EPAS1*, have provided insights into complex histories of ancient introgression, including examples of admixture from now-extinct source populations. In Tibetan canids and Andean waterfowl, directed mutagenesis experiments involving introgressed hemoglobin variants successfully identified causative amino acid mutations and characterized their phenotypic effects, thereby providing insights into the functional properties of selectively introgressed alleles. We review case studies of adaptive introgression in high-altitude vertebrates and we highlight findings that may be of general significance for understanding mechanisms of environmental adaptation involving different sources of genetic variation.

## Introduction

Adaptation to changing environmental conditions has traditionally been viewed as a process that is fueled by two principal sources of genetic variation: newly arisen mutations or pre-existing allelic variants (standing variation). However, recent genomic studies of animal populations have revealed numerous cases in which putatively adaptive changes are attributable to allelic variants that were introduced via hybridization with a closely related species (Hedrick, 2013; Racimo et al., 2017; Taylor and Larson, 2019). Several especially well-documented examples of adaptive introgression involve humans and other vertebrates native to high altitude. In such studies, results of adaptive introgression can be examined in a well-defined ecological context where evolutionary responses to hypoxia and other altitude-related environmental challenges can be understood and interpreted in terms of specific physiological phenotypes. Case studies involving high-altitude vertebrates may therefore provide generalizable insights into the process of adaptive introgression and the functional attributes of introgressed alleles.

When a lowland species colonizes a highland environment, numerous physiological traits may be subject to novel selection pressures owing to the lower partial pressure of O_2_ (*P*O_2_), lower air temperature, and other environmental changes. Indeed, comparative studies of high-altitude vertebrates have revealed consistent patterns of evolutionary change in respiratory, cardiovascular, and metabolic traits that contribute to hypoxia tolerance (Storz et al., 2010b, 2019; Ivy and Scott, 2015; McClelland and Scott, 2019; Storz and Scott, 2019). Initially, the response to selection on such traits is based on standing variation within the colonizing population and/or the input of new mutations. In principle, however, adaptation to newly encountered environmental challenges may be facilitated by hybridization with a closely related resident species that has already evolved solutions to such challenges. In such cases, the process of adaptation may be greatly expedited because the newly arrived recipient species can capitalize on adaptive solutions that have been refined over a much longer period of time in the donor species.

Population genomic studies of high-altitude vertebrates have implicated numerous genes in hypoxia adaptation, chief among them are components of the hypoxia-inducible factor (HIF) pathway and hemoglobin (Hb) genes (Schweizer et al., 2019; Witt and Huerta-Sánchez, 2019; Storz, 2021; Storz and Cheviron, 2021). These same genes figure prominently in documented cases of adaptive introgression in high-altitude populations. Case studies involving the HIF pathway have focused on *EPAS1* (*endothelial PAS domain containing protein 1*), a gene that encodes the O_2_-regulated subunit of the dimeric HIF-2α transcription factor. The *EPAS1* studies involved genomic analyses and have provided insights into complex histories of relatively recent admixture as well as ancient introgression (Huerta-Sánchez et al., 2014; Jeong et al., 2014; Huerta-Sánchez and Casey, 2015; Hu et al., 2017; Miao et al., 2017; vonHoldt et al., 2017; Wang et al., 2020). Case studies involving Hb variants successfully identified causative amino acid mutations and the functional and structural mechanisms by which they exert their phenotypic effects (Natarajan et al., 2015; Signore et al., 2019), thereby providing insights into the functional properties of adaptively introgressed allelic variants. Below we summarize results of these studies and discuss insights that inform our understanding of adaptive introgression.

### *EPAS1* in Tibetan Humans

*EPAS1* has been implicated as a candidate gene for hypoxia adaptation in Tibetan humans (Beall et al., 2010; Simonson et al., 2010; Yi et al., 2010) and several other highland vertebrates (Witt and Huerta-Sánchez, 2019; Storz, 2021; Storz and Cheviron, 2021). In Tibetan highlanders, derived variants in intronic regions of *EPAS1* are present at much higher frequencies than in closely related lowland groups such as the Han Chinese, and are associated with low (non-elevated) blood Hb concentration, a characteristically Tibetan hematological phenotype. In Tibetans living at elevations >4,000 m above sea level, average Hb concentration is only slightly higher than normal values expected for people living at sea level (Beall et al., 1998). Since Hb concentration typically increases during acclimatization to environmental hypoxia, the observed Hb concentrations of Tibetan highlanders are “low” in a relative sense, in comparison with values for other native highlanders (e.g., Quechua and Aymara highlanders in the Andes) and acclimatized lowlanders living at similar altitudes. Since Hb concentration is a highly plastic phenotype that is responsive to cellular hypoxia, it is not clear whether the observed association with *EPAS1* genotype reflects a causal relationship or whether it is an indirect consequence of induced changes in other traits (Storz, 2021; Storz and Cheviron, 2021). Although the population-genetic evidence for positive selection on *EPAS1* is extremely compelling, the direct phenotypic effects of the variants have yet to elucidated.

**Figure 1:**
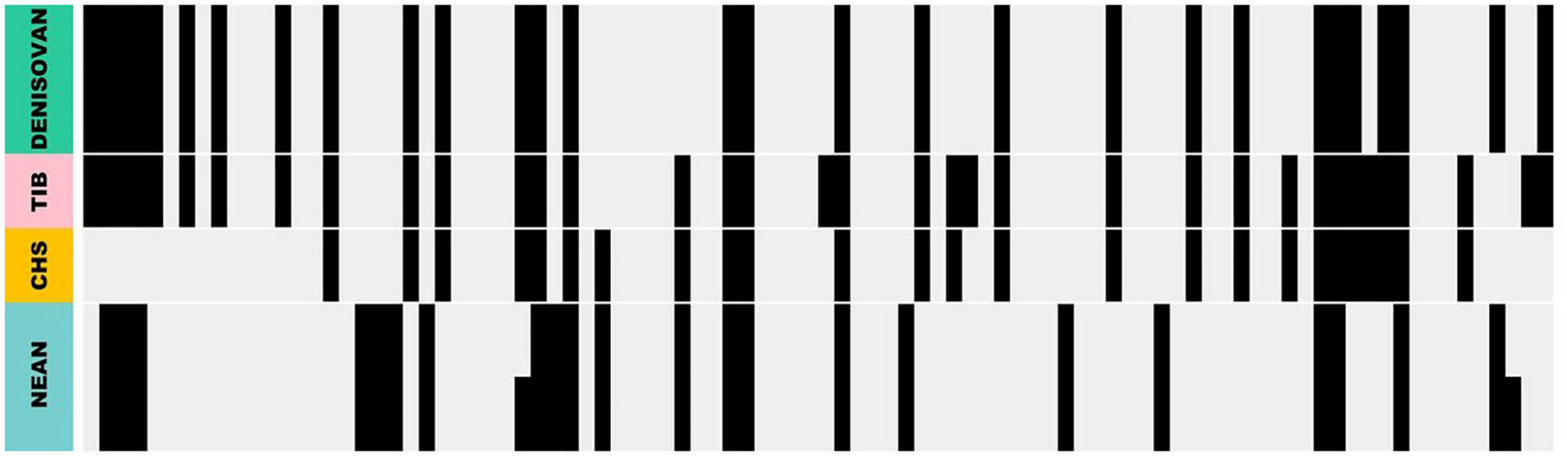
Comparison with Denisovan and Neanderthal haplotypes. Each column represents a single nucleotide polymorphism (SNP). The first two rows are the two Denisovan haplotypes (in green), followed by the Tibetan (TIB) most common haplotype (in pink), the modern human haplotype in the 1,000 Genomes Project dataset that exhibits the closest match to the Tibetan haplotype [in yellow, Han Chinese (CHS)], and the two Neanderthal (NEAN) haplotypes (in blue). Derived (nonancestal) nucleotide states are shown in black. At any given site, a nucleotide state is considered derived if it differs from the corresponding state in the orthologous chimpanzee sequence, and it is considered ancestral if it matches the state in chimpanzee. Reproduced from Huerta-Sánchez and Casey (Huerta-Sánchez and Casey, 2015) with permission.

Population genetic analysis of *EPAS1* polymorphism in Tibetans revealed one of the most striking signatures of positive selection ever documented in humans (Yi et al., 2010), but the pattern of nucleotide variation could not be easily reconciled with models of selection on standing variation or new mutations (Huerta-Sánchez et al., 2014). Re-sequencing of a ~130 kb chromosomal region that spans the entire coding region of *EPAS1* revealed a 32.7 kb segment that exhibited an exceedingly high level of nucleotide divergence between Tibetan and Han Chinese haplotypes, and many derived nucleotide states in the Tibetan haplotype were not shared with *EPAS1* sequences sampled from a globally diverse set of other populations (Peng et al., 2011; Huerta-Sánchez et al., 2014; Hu et al., 2017). Remarkably, a broader comparative analysis revealed that the Tibetan haplotype exhibited a close match to orthologous sequence from Denisovans (Huerta-Sánchez et al., 2014) (Figure 1), an extinct hominin group that is more closely related to Neanderthals than to modern humans. Genomic analyses clearly demonstrate that the highly divergent *EPAS1* haplotype of Tibetans was derived via introgression from Denisovans, thereby explaining the anomalous patterns of nucleotide variation (Huerta-Sánchez et al., 2014; Hackinger et al., 2016; Hu et al., 2017).

Until recently, the only fossil material for Denisovans came from a single site in southern Siberia, Denisova Cave, and everything else known about the historical geographic range of this group is inferred from traces of Denisovan ancestry in the genomes of modern humans. Denisova Cave is located at only 700 m elevation, and the largest shares of Denisovan ancestry have been identified in the genomes of lowland Asians, indigenous Australians, and Melanesians (Reich et al., 2010, 2011; Mallick et al., 2016; Sankararaman et al., 2016; Browning et al., 2018; Jacobs et al., 2019; Teixeira et al., 2021). The possession of an apparently “altitude-adapted” *EPAS1* haplotype in Denisovans therefore seemed difficult to reconcile with available evidence for a predominantly lowland distribution. However, the recent discovery of Denisovan fossil material and DNA from a karst cave at 3,280 m on the Tibetan Plateau provides evidence that this enigmatic group inhabited this high-altitude region during the Middle Pleistocene epoch (Chen et al., 2019; Zhang D. J. et al., 2020) and therefore could have adapted to life at high altitude long before the arrival of anatomically modern humans.

The discovery that the Tibetan *EPAS1* haplotype is derived from Denisovans, in combination with the discovery that Denisovans inhabited high altitudes on the Tibetan Plateau, prompts additional questions: Are Tibetan genomes disproportionately enriched for introgressed Denisovan DNA compared to those of lowland groups? Did introgression from Denisovans contribute to other aspects of high-altitude adaptation? With regard to the first question, genomic analyses indicate that the proportion of Denisovan admixture in Tibetan highlanders is 0.4% (95% confidence interval = 0.2–0.6%) (Hu et al., 2017), similar to that estimated for Han Chinese and other lowland Asian groups (Mallick et al., 2016, Sankararaman et al., 2016). With regard to the second question, a thorough analysis of genomic polymorphism data in Tibetan highlanders revealed that *EPAS1* is the only locus in Denisovan-derived introgression tracts that exhibits statistical evidence for positive selection (Hu et al., 2017; Zhang X. et al., 2020). Thus, Tibetans do not show evidence for disproportionate admixture with Densiovans and—aside from *EPAS1*—there is no evidence that such admixture contributed to high-altitude adaptation. Results of genomic analyses also suggest that introgression of the Denisovan *EPAS1* haplotype occurred in the common ancestor of the Tibetan and Han populations (Hu et al., 2017), and that the introgression occurred prior to the onset of positive selection in Tibetan highlanders, consistent with a scenario of adaptation via selection on standing archaic variation (Zhang X. et al., 2020).

### *EPAS1* in Tibetan Canids

Similar to the case with Tibetan humans, population genomic analyses of wolves and dogs from high-altitude regions of the Tibetan Plateau and Himalaya revealed clear evidence for positive selection on *EPAS1* (Gou et al., 2014; Wang et al., 2014; Zhang et al., 2014). Subsequent genomic analyses of high-altitude canids suggested that the sharing of *EPAS1* haplotypes between Tibetan wolf and Tibetan mastiff (a highly distinctive, highland dog breed) is attributable to a history of introgressive hybridization (Miao et al., 2017; vonHoldt et al., 2017). It was originally assumed that a pre-existing *EPAS1* variant in Tibetan wolf (the long-term resident) introgressed into Tibetan mastiff (the relatively recent arrival) at some point during the Paleolithic Age when humans and their commensal animals first settled the Tibetan Plateau. A comparative population genomic analysis revealed a surprisingly complex history of admixture and introgression between high-altitude wolves and dogs, and indicated that the genomes of high-altitude wolves reflects a history of ancient admixture with a now-extinct wolf-like canid. This deeply divergent “ghost” lineage appears to be the source of the altitude-adapted *EPAS1* allele (Wang et al., 2020), lending credence to the idea that the allele existed long before the arrival of domestic dogs on the Tibetan Plateau.

### *β-globin* in Tibetan Canids

In addition to the *EPAS1* gene region, genomic studies of Tibetan mastiffs revealed a striking signature of positive selection in the chromosomal region spanning the β-globin gene cluster (Gou et al., 2014; Wang et al., 2014), a compact set of tandemly duplicated genes that encode the β-type subunits of pre- and postnatally expressed Hb isoforms (Storz, 2016a). Although all canids possess three paralogous adult β-type globin genes (5′-*HBD*-*HBB/D*-*HBB*-3′), the β-chain of the major adult Hb isoform is encoded by *HBB/D*, a chimeric fusion gene comprising the 5′ portion of the parental *HBD* gene (δ-globin) and the 3′ portion of the parental *HBB* gene (β-globin) (Gaudry et al., 2014; Signore et al., 2019). The search for potentially selected sites in the β-type globin genes of Tibetan mastiff revealed that the *HBB/D* gene of this breed is distinguished from that of all other domestic dog breeds by just two missense mutations at adjacent codons: β13Gly→Ser and β14Leu→Met. In addition to evidence for a selective sweep spanning the β-globin gene cluster of Tibetan mastiff, comparative population genomic analyses revealed that the entire cluster was introgressed from the Tibetan wolf (Miao et al., 2017). Thus, the adult Hbs of Tibetan mastiff and Tibetan wolf are distinguished from those of all other dogs and wolves by the same pair of amino acid replacements at β13 and β14. The evidence for positive selection on the wolf-derived β-globin variants in Tibetan mastiff suggests that the introgression contributed to hypoxia adaptation at high altitude.

**Figure 2:**
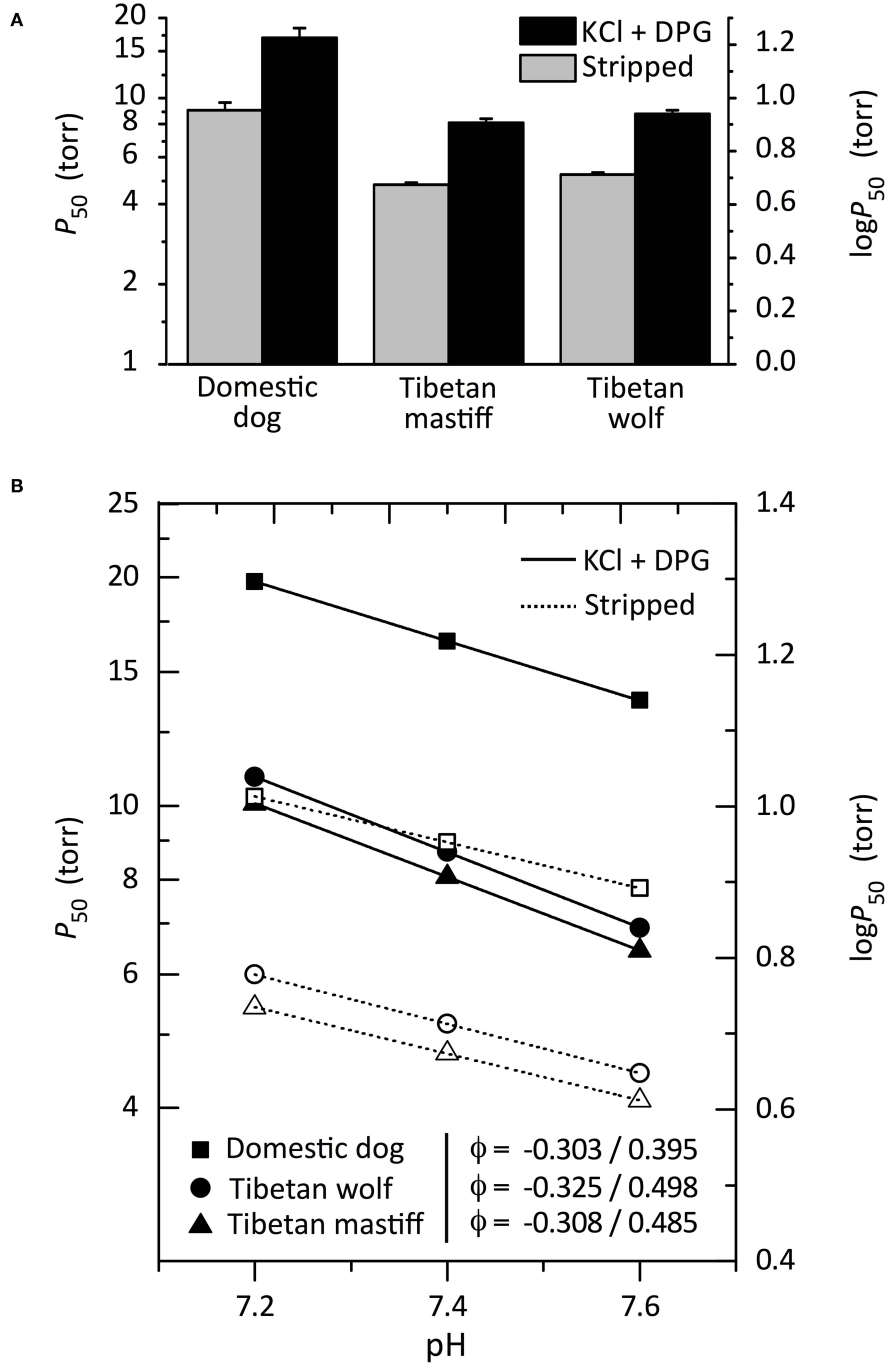
Oxygenation properties of purified Hbs from Tibetan wolves, Tibetan mastiffs, and domestic dogs in the absence (“stripped”) and presence of allosteric cofactors [Cl^−^ ions (KCl) and 2,3-diphosphoglycerate (DPG)]. **(A)** *P*_50_-values (±SEM), the *P*O_2_ at 50% saturation, at 37°C, pH 7.4. The lower the *P*_50_, the higher the O_2_-affinity. **(B)** Bohr effect of canid Hbs, as indicated by a plot of log-*P*_50_ vs. pH in the presence and absence of allosteric cofactors (filled and open symbols, respectively). Bohr coefficients (φ) are shown in the absence/presence of allosteric cofactors. Reproduced from Signore et al. (2019).

In the absence of compensatory physiological adjustments at high altitude, the reduced *P*O_2_ of inspired air translates into a reduction in arterial O_2_ content (hypoxemia), thereby impairing O_2_ flux to metabolizing tissues. Under conditions of severe hypoxia, an increased Hb-O_2_ affinity can help safeguard arterial O_2_ saturation, thereby minimizing the inevitable reduction in tissue O_2_ delivery (Storz, 2016b, 2019). Evolved increases in Hb-O_2_ affinity and—in some cases—associated increases in arterial O_2_ saturation in hypoxia, have been documented in several highly aerobic mammals that are native to high altitude (Storz et al., 2010a; Tate et al., 2017; Ivy et al., 2020; Signore and Storz, 2020). In the case of Tibetan mastiff Hb, a clear hypothesis is that the introgressed, wolf-derived *HBB/D* variants are adaptive at high altitude because they confer an increased Hb-O_2_ affinity. To test this hypothesis, Signore et al. (2019) experimentally characterized the oxygenation properties of purified native Hbs from Tibetan wolves and Tibetan mastiffs (which both share the derived genotype, β13-Ser/β14-Met), and other breeds of domestic dog (which—along with gray wolves—possess the ancestral genotype, β13-Gly/β14-Leu). This comparison revealed the net effect of the two amino acid replacements at β13/β14, as the canid Hbs are otherwise structurally identical. Hbs of Tibetan wolf and Tibetan mastiff have a significantly higher intrinsic O_2_-affinity [measured as *P*_50(stripped)_, the *P*O_2_ at which Hb is 50% saturated] relative to the Hbs of lowland domestic dogs (Figure 2A). In the presence of allosteric cofactors such as chloride ions and organic phosphates [2,3-diphosphoglycerate (DPG)], O_2_-affinities of all canid Hbs were reduced to the same relative extent (Figure 2A), indicating that the observed difference in Hb-O_2_ affinity is purely attributable to an evolved change in intrinsic heme reactivity. Hbs of Tibetan wolf and Tibetan mastiff also exhibited a significantly higher Bohr effect (i.e., a higher sensitivity to pH) relative to the Hbs of all domestic dogs (Figure 2B). The Bohr effect describes how Hb-O_2_ affinity changes as an inverse function of pH, which allows more complete O_2_ unloading to acidic tissues during exercise. Since an increased Hb-O_2_ affinity can impede O_2_ unloading in the systemic circulation, the enhanced pH-sensitivity of Tibetan canid Hbs should compensate by promoting O_2_ unloading to working muscle.

**Figure 3:**
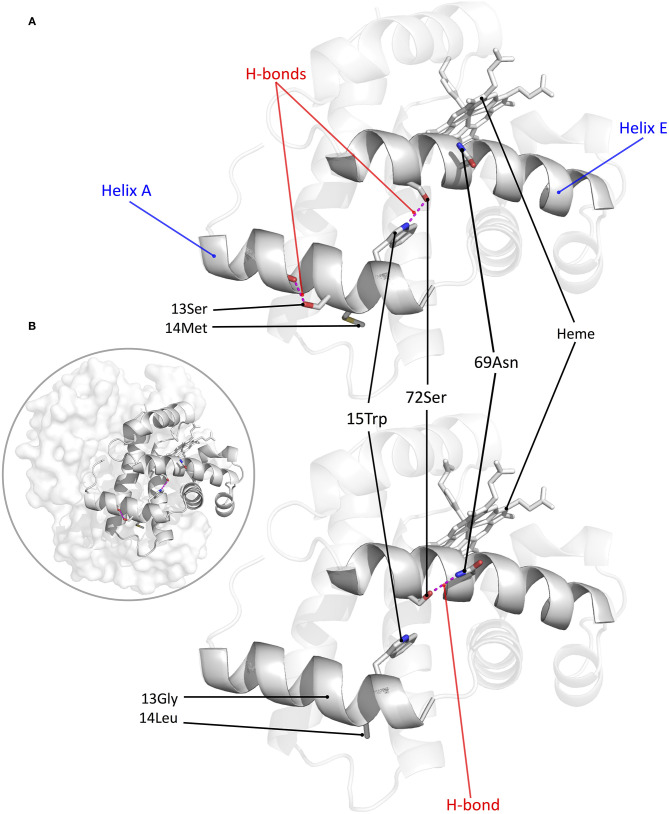
Structural model of canine Hb showing the net effect of β-chain substitutions G13S and L14M. **(A)** β-chain of Tibetan mastiff/Tibetan wolf Hb, showing that the two substitutions result in the addition of two intra- and inter-helical hydrogen bonds (9-Ser: 13-Ser and 15-Trp: 72-Ser, respectively). **(B)** β-chain of domestic dog/gray wolf Hb, showing that 72-Ser forms an intrahelical hydrogen bond with 69-Asn instead of the interhelical bond with 15-Trp. Inset: structural model of tetrameric canine Hb with the β-chain subunit highlighted.

**Figure 4:**
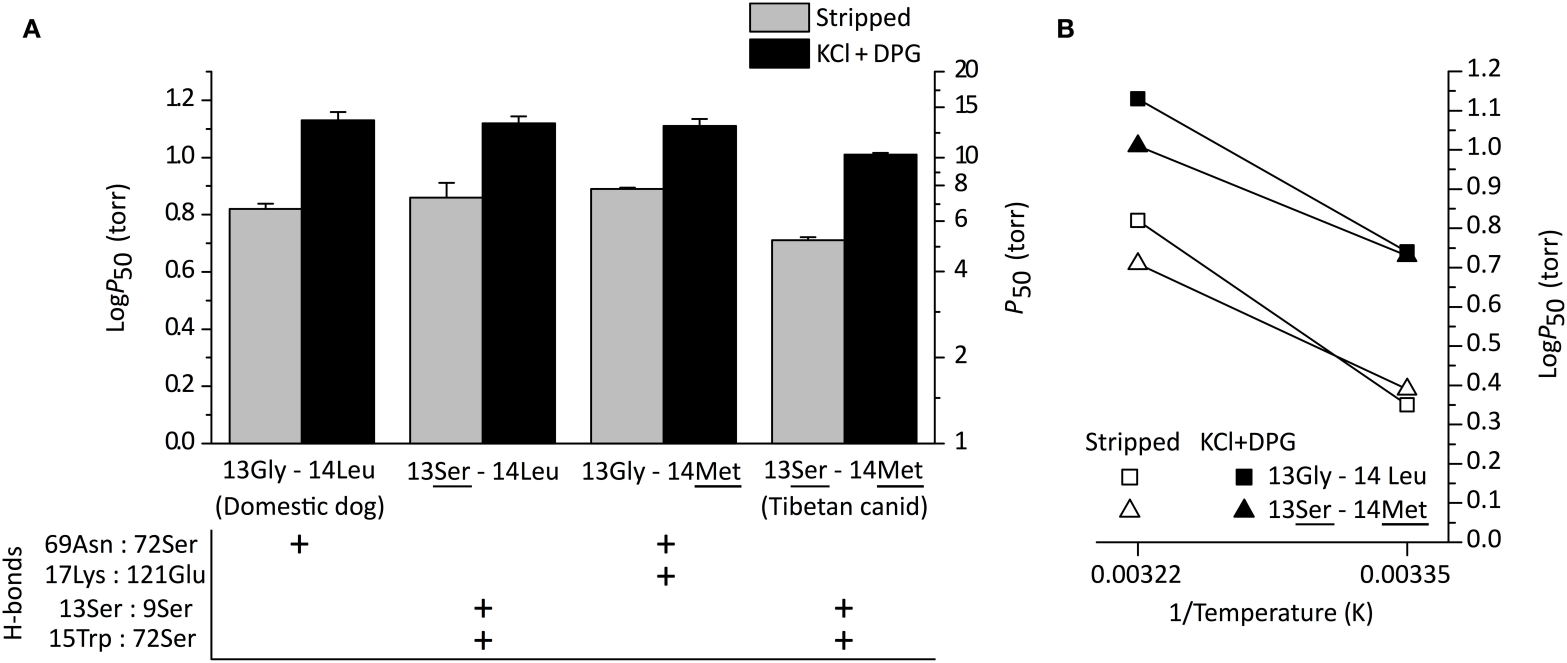
Functional effects of amino acid replacements in Tibetan mastiff and Tibetan wolf Hb. **(A)** O_2_-affinities of recombinant Hbs representing the (ancestral) wildtype genotype of domestic dog/gray wolf (13Gly-14Leu), the derived, double-mutant genotype of Tibetan mastiff/Tibetan wolf (13**Ser**-14**Met**), and each of the possible single-mutant genotypes (13**Ser**-14Leu and 13Gly-14**Met**). *P*_50_-values (± SEM) are shown in the absence (“stripped”) and presence of allosteric effectors (“KCl+DPG”) at 37°C. The lower the *P*_50_, the higher the O_2_ affinity. *P*_50_-values for the double-mutant (13**Ser**-14**Met**) were significantly higher than those of the other three genotypes for both treatments (stripped and KCl+DPG). For each genotype, the panel below denotes the presence and absence of key hydrogen bonds in the β-chain subunit. **(B)** van't Hoff plot of rHbs representing wildtype genotypes of domestic dog/gray wolf (13Gly-14Leu) and Tibetan mastiff/Tibetan wolf (13**Ser**-14**Met**) derived from O_2_-equilibrium curves measured at 25 and 37°C. Reproduced from Signore et al. (2019).

Structural modeling of canine Hb revealed that derived amino acid replacements found in Tibetan wolf and Tibetan mastiff (β13Gly→Ser and β14Leu→Met) result in a rearrangement of hydrogen bonds within and between α-helices of the Hb β-chain (Signore et al., 2019) (Figure 3). This rearrangement of hydrogen bonds (including the net addition of two new hydrogen bonds per tetramer) is predicted to increase heme reactivity by stabilizing the high-affinity, oxygenated conformation of the Hb tetramer (the “R” state) relative to the low-affinity, deoxy conformation (the “T” state). Modeling results predicted that both replacements at β13 and β14 are necessary to produce the observed increase in O_2_-affinity, and that either mutation by itself would be insufficient. To test these predictions, Signore et al. (2019) synthesized and experimentally tested four recombinant Hb (rHb) mutants representing each possible two-site combination of ancestral/derived amino acid states at β13 and β14. Consistent with model-based predictions, experimental measurements of the four rHbs revealed that mutations at the two focal sites only produce a significant increase in Hb-O_2_ affinity in combination (Figure 4A). To test the structural hypothesis that the increase in Hb-O_2_ affinity is attributable to a T→ R shift in the conformational equilibrium, Signore et al. (2019) measured the thermodynamics of heme oxygenation and quantified the difference in the enthalpy of oxygenation (ΔΔ*H*) between the double mutant 13Ser-14Met genotype (characteristic of Tibetan canids) and the 13Gly-14Leu genotype (characteristic of domestic dogs and gray wolves). Measurements in the absence of allosteric effectors (“stripped”) revealed that the Tibetan canid rHb exhibited a numerically lower Δ*H*′ relative to that of domestic dog/gray wolf (as indicated by the lower slope of the van't Hoff plot in Figure 4B). The measured difference in Δ*H*′ reflects the change in enthalpy caused by the breakage/formation of bonds in the oxygenation-linked T→R transition (Δ*H*^T→*R*^), consistent with the model-based prediction. In summary, the protein engineering experiments identified causative mutations that underlie a putatively adaptive change in protein function and characterized the functional and biophysical mechanisms by which they exert their effects on a physiologically important and adaptively relevant blood phenotype.

### *β-globin* in Andean Waterfowl

**Figure 5:**
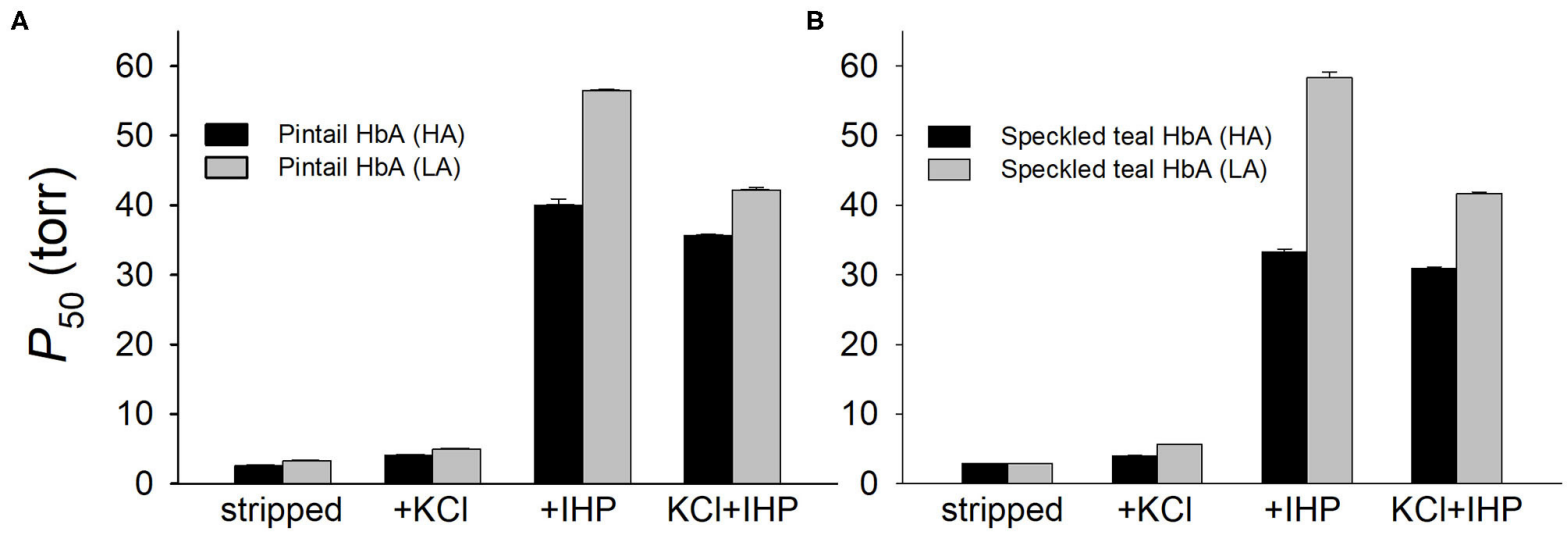
Oxygenation properties of the major HbA isoforms from high- and low-altitude populations of **(A)** yellow-billed pintail, *Anas georgica*, and **(B)** and speckled teal, *Anas flavirostris*. In both species, alternative HbA variants that predominate in high- and low-altitude populations differ at two sites, β^*A*^116 and β^*A*^133. HA, high-altitude; LA, low-altitude. *P*_50_-values (means ± SEM) of purified HbA were measured at pH 7.4 and 37°C in the absence (stripped) and presence of allosteric effectors: chloride ions (added as KCl) and inositol hexaphosphate (IHP) ([Cl^−^], 0.1 M; [HEPES], 0.1 M; IHP/Hb tetramer ratio, 2.0; [Heme], 0.300 mM). Based on data in Natarajan et al. (2015).

Many high-altitude Andean waterfowl have convergently evolved increased Hb-O_2_ affinities in comparison to lowland conspecifics and lowland sister species (Natarajan et al., 2015), a consistent pattern of convergence in highland birds (Projecto-Garcia et al., 2013; Galen et al., 2015; Natarajan et al., 2016, 2018; Storz, 2016b; Kumar et al., 2017; Jendroszek et al., 2018; Zhu et al., 2018). In the Andes, highland populations of two broadly distributed species, yellow-billed pintail (*Anas georgica*) and speckled teal (*Anas flavirostris*), both exhibit higher Hb-O_2_ affinities relative to lowland populations of the same species (Figure 5). Experiments on purified native Hbs of both species revealed that altitude-related differences in Hb function are attributable to the independent or joint effects of amino acid replacements at two β-chain sites: β116Ala→Ser, β133Leu→Met (in both cases, the highland amino acid variants are derived). The affinity-enhancing effects were manifest in the major and minor Hb isoforms (HbA and HbD, respectively) since both isoforms incorporate the same β-chain subunit.

In both species, experimental appraisals of Hb function were combined with population genomic analyses to test for corroborative evidence that the affinity-enhancing amino acid variants had increased in frequency in the high-altitude populations under the influence of positive selection (Natarajan et al., 2015). In the comparison between high- and low-altitude populations of yellow-billed pintail, the mean site-specific differentiation in allele frequency (*F*_ST_) across the genome was 0.02, whereas that for β116/β133 was 0.90 [far exceeding the 99th percentile of the genome-wide distribution (=0.26)]. In comparisons between high- and low-altitude populations of speckled teal, the average genome-wide *F*_ST_ was 0.06, whereas that for β116/β133 was 1.00 [the maximal value, indicating that alternative alleles were fixed in the high- and low-altitude populations, far exceeding the 99th percentile of the genome-wide distribution (=0.42)]. In addition to this population genomic evidence for altitude-related selection on the linked β-globin variants, phylogenetic analysis clearly revealed that the shared 116Ser-133Met alleles in both species are identical-by-descent, and that the 116Ser-133Met allele in high-altitude yellow-billed pintails was derived via introgression from high-altitude speckled teals (Natarajan et al., 2015). Hybridization between these two species has been documented previously (McCracken and Wilson, 2011). Similar to other cases of adaptive introgression involving Tibetan humans and Tibetan canids, the waterfowl example involves a donor species (speckled teal) that appears to have a longer history of residence at high-altitude relative to the recipient species (yellow-billed pintail).

**Figure 6:**
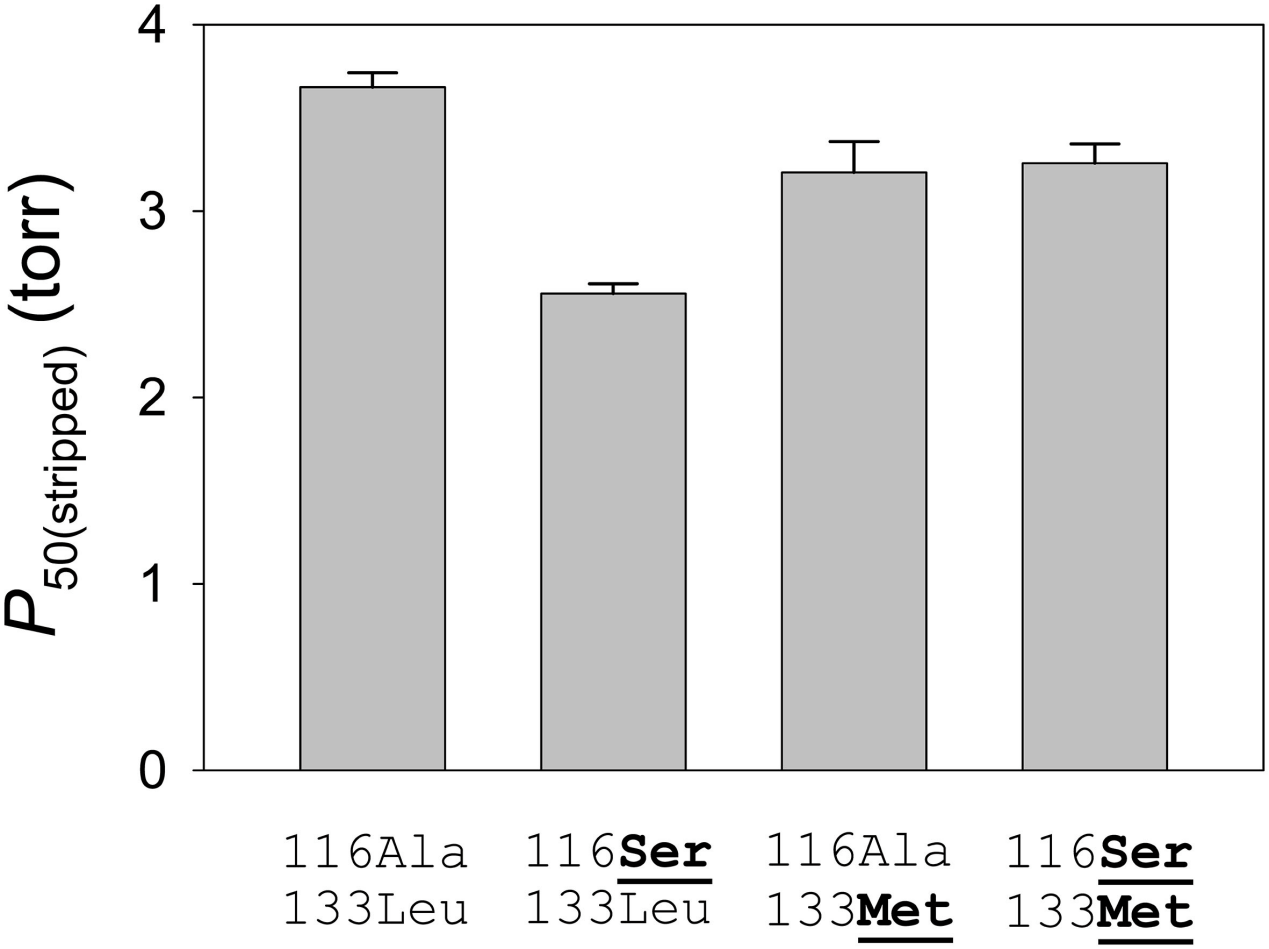
Intrinsic O_2_-affinities [*P*_50(stripped)_, torr; mean ± SEM] of purified yellow-billed pintail rHb mutants measured in the absence of allosteric effectors. O_2_-equilibrium curves for each rHb mutant were measured in 0.1 M HEPES buffer at pH 7.40, 37°C; [heme], 0.3 mM. Numbers refer to residue positions in the β-chain subunit. “116Ala-133Leu” and “116Ser-133Met” are the two-site genotypes that predominate in low- and high-altitude populations, respectively. At each site, the derived (non-ancestral) amino acids are underlined in bold. Reproduced from Natarajan et al. (2015).

To measure the independent and joint effects of the β116Ala→ Ser and β133Leu→ Met replacements on a standardized genetic background, Natarajan et al. (2015) used site-directed mutagenesis to engineer recombinant yellow-billed pintail HbA mutants representing each of four possible two-site genotypic combinations: the wildtype low-altitude genotype (116Ala-133Leu, which represents the ancestral low-altitude state), the derived, double-mutant high-altitude genotype (116Ser-133Met), and each of the two possible single-mutant intermediates (116Ser-133Leu and 116Ala-133Met). Experimental measurements of the rHb mutants recapitulated the observed difference in intrinsic O_2_-affinity between the HbA variants of high- and low-altitude yellow-billed pintails, and revealed that the observed difference is mainly attributable to β116Ala→ Ser (Figure 6). On the ancestral background (116Ala-133Leu), the β116Ala→ Ser mutation produced a 16% reduction in *P*_50_ in the presence of physiological concentrations of allosteric effectors [Cl^−^ ions and the organic phosphate, inositol hexaphosphate (IHP)], the experimental treatment most relevant to *in vivo* conditions in the red blood cell.

**Figure 7:**
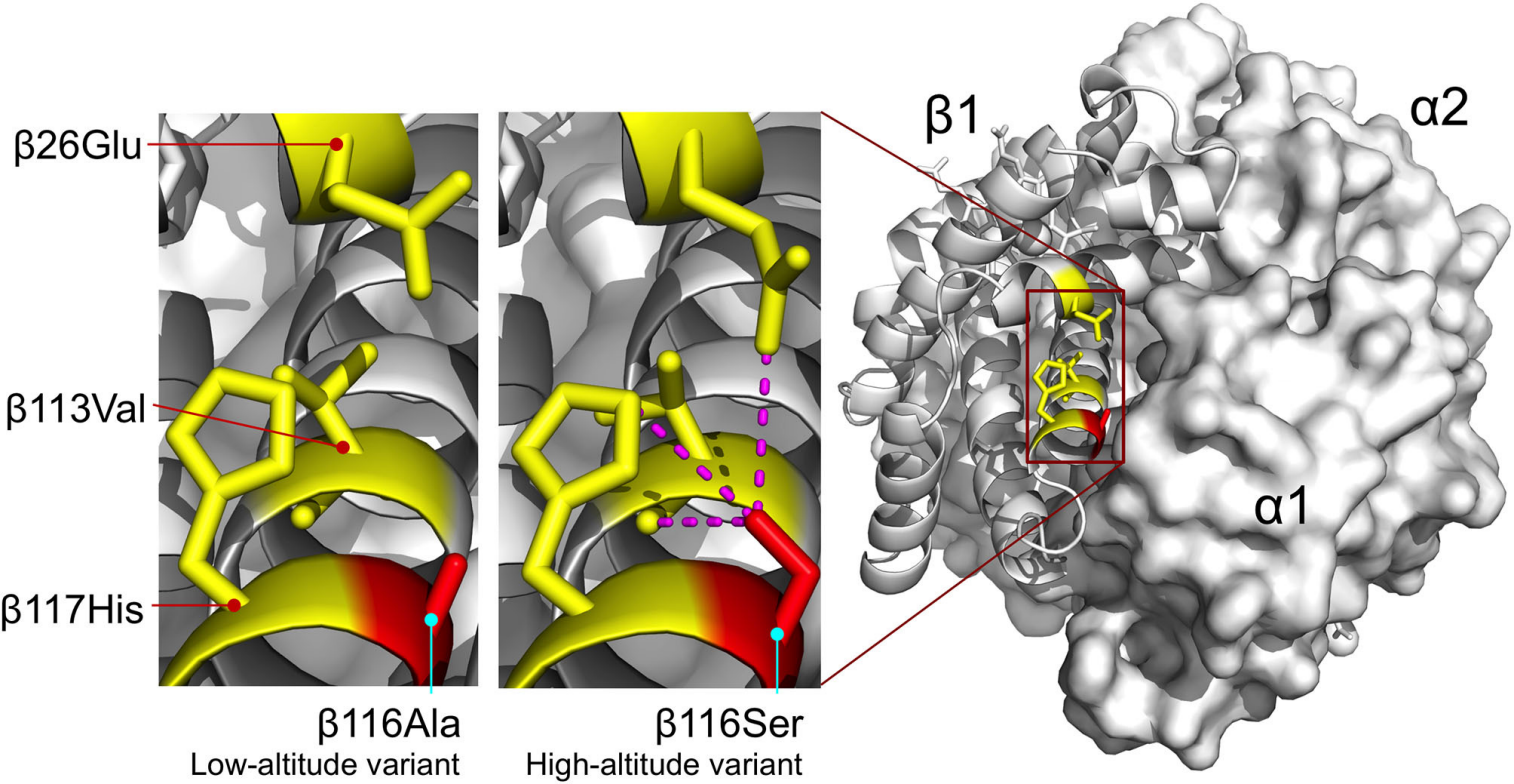
Homology model of yellow-billed pintail HbA showing the location of an affinity-enhancing β116Ala→Ser replacement that distinguishes high- and low-altitude variants. Replacement of the nonpolar Ala for an uncharged, polar Ser at the α1β1 intradimer contact surface is predicted to increase Hb-O_2_ affinity by stabilizing the R-state via intra-subunit hydrogen bonds between the γ-oxygen of β116Ser and each of three β-chain residues: β26Glu (ε2-oxygen), β113Val (carbonyl oxygen), and β117His (ε2-nitrogen). Homology modeling indicates that the same network of interhelical bonds is not present in the T-state. Reproduced from Natarajan et al. (2015).

The β116Ala→ Ser substitution involves the replacement of nonpolar alanine for an uncharged, polar serine at an α1β1 intradimer contact. This replacement appears to increase O_2_-affinity by stabilizing the R-state via intra-subunit hydrogen bonds between the γ-oxygen of β116Ser and each of three β-chain residues: β26Glu (ε2-oxygen), β113Val (carbonyl oxygen), and β117His (ε2-nitrogen) (Figure 6). Similar to the effect described for the Hbs of Tibetan canids, the increase in O_2_ affinity of the duck Hbs is achieved via stabilization of the R-state rather than destabilization of the T-state (Jendroszek et al., 2018; Storz, 2019). Also similar to the case with Tibetan canids, there is a nonadditive interaction between two affinity-altering mutations (Figure 7), although the biophysical mechanism underlying the interaction effect in the duck Hbs is not clear.

## Conclusions

The *EPAS1* case studies of high-altitude humans and canids illustrate complex histories of introgression including admixture from now-extinct source populations. In broad outline, results appear to be consistent with the hypothetical scenario of introgression involving a long-term highland species as the donor and a more recently arrived colonizing species as the recipient. However, in the case of the canids, the highly admixed ancestries of high-altitude wolves and dogs make it difficult to draw firm conclusions about the relationships among the hybridizing taxa and the timing of introgression events. Although the evidence for positive selection on *EPAS1* is compelling in both humans and canids, the adaptive mechanism and the phenotypic target of selection have yet to be fully elucidated. In Tibetan humans, derived nucleotide changes in the introgressed *EPAS1* allele are concentrated in introns, so causal mutations likely affect transcriptional regulation of the gene. In Tibetan canids, introgressed *EPAS1* alleles harbor both coding and noncoding mutations. Insights regarding adaptive mechanisms are much more clear in the Hb studies, as these provide the only cases where causative mutations have been identified and functionally characterized via site-directed mutagenesis experiments.

In contrast to de novo mutations and low-frequency variants, introgressed alleles that were previously fixed in a different species have been pre-tested by selection (albeit on a different genetic background). It has therefore been suggested that selectively introgressed alleles may be especially likely to combine multiple mutations (e.g., epistatic modifiers) that interact to fine-tune a selected phenotype and/or mitigate deleterious pleiotropic effects of function-altering mutations (Hedrick, 2013). It is therefore especially noteworthy that the increased O_2_-affinity of Tibetan canid Hb required both mutations at β13 and β14 in combination, so neither mutation by itself would have been sufficient to confer an adaptive benefit at high altitude. Additional comparative data are needed to assess whether the mechanistic underpinnings of Hb adaptations derived from introgression are qualitatively distinct from adaptive changes in Hb function that evolved *via* the sequential fixation of *de novo* mutations and/or segregating variants.

Sophisticated statistical approaches have been developed that use genomic polymorphism data to identify introgression tracts and to detect evidence of positive selection on introgressed variants. However, what is typically missing is experimental evidence that an introgressed allelic variant has contributed to increased fitness on the genetic background of the recipient species, or that it causes a change in phenotype in the direction that is predicted to be adaptive. As stated by Taylor and Larson (2019, 173), “…it is necessary to demonstrate an adaptive function for the introgressed genomic regions before claiming the discovery of adaptive introgression.” Future studies of introgressive hybridization in high-altitude species that combine genomic data with mechanistic approaches in experimental physiology should play an important role in advancing our understanding of the role of introgression in environmental adaptation.

## Author Contributions

All authors listed have made a substantial, direct and intellectual contribution to the work, and approved it for publication.

## Conflict of Interest

The authors declare that the research was conducted in the absence of any commercial or financial relationships that could be construed as a potential conflict of interest.
